# Bonding Efficacy of Universal Resin Adhesives to Zirconia Substrates: Systematic Review and Meta-Analysis

**DOI:** 10.3290/j.jad.b3868649

**Published:** 2023-02-06

**Authors:** Renally Bezerra Wanderley Lima, Aline Fedoce Silva, Wellington Luiz de Oliveira da Rosa, Evandro Piva, Rosângela Marques Duarte, Grace Mendonça De Souza

**Affiliations:** a Assistant Professor, Department of Dentistry, Nova Esperança Faculty (FACENE), João Pessoa, PB, Brazil. Conceptualization, methodology, formal analysis, data curation, wrote the original draft.; b Assistant Professor, School of Dentistry, Federal University of Juiz de Fora, MG, Brazil. Methodology, investigation, data curation, wrote the original draft.; c Assistant Professor, Department of Restorative Dentistry, School of Dentistry, Federal University of Pelotas (UFPEL), Pelotas, RS, Brazil. Data curation, wrote, reviewed and edited the manuscript.; d Professor, Department of Restorative Dentistry, School of Dentistry, Federal University of Pelotas (UFPEL), Pelotas, RS, Brazil. Data curation, wrote, reviewed and edited the manuscript.; e Professor, Department of Restorative Dentistry, Federal University of Paraiba (UFPB), João Pessoa, PB, Brazil. Conceptualization, study supervisor, formal analysis, wrote, reviewed and edited the manuscript.; f Associate Professor, Department of Comprehensive Dentistry, University Louisville, Louisville, KY, USA. Conceptualization, study supervisor, formal analysis, wrote, reviewed and edited the manuscript.

**Keywords:** adhesives, all ceramics, bond strength, prosthodontic ceramics.

## Abstract

**Purpose:**

To provide an overview of the in-vitro bond strength of universal adhesives to zirconia and analyze whether these adhesives are a reliable alternative to conventional zirconia primers.

**Materials and Methods:**

A systematic search was conducted in PubMed/Medline, Scopus, and ISI Web of Science databases up to August 2021. Investigations published in English, assessing resin-mediated bond to zirconia using universal adhesives compared to phosphate/silane-based primer or phosphate-based primer were included. After study selection and data extraction, risk of bias analysis was performed. Statistical analyses were performed using RevMan 5.4, with a random effects model, at a significance level of 0.05.

**Results:**

In total, 23 studies were included for qualitative and quantitative analysis. Universal adhesives showed higher bond strengths than did phosphate-based primers (p < 0.00001) to aged zirconia without airborne alumina-particle abrasion. Similar results were observed when the zirconia surface was airborne-particle abraded at baseline and after dynamic aging (p < 0.0001). When universal adhesives and phosphate-silane based primers were compared, similar bond strengths (p ≥ 0.001) were observed after surface abrasion, regardless of storage condition.

**Conclusion:**

The results showed that universal adhesives generate higher bond strengths when compared to conventional zirconia primers.

Yttria-stabilized tetragonal zirconia polycrystalline (Y-TZP) is the most commonly used zirconia in dentistry. Chemical bonding to Y-TZP has been a challenge for clinicians because of its high crystallinity and chemical inertness.^39,58^ In clinical situations where only micromechanical retention is needed, zirconia restorations can be bonded to the abutment tooth and restorative material using conventional cements. However, reliable resin-mediated bonding to zirconia is required in addition to micromechanical interlocking in the case of non-retentive preparations. Resin-mediated bonding to zirconia is often based on chemical bonding through conditioning with a primer.^22,44,58^ Using an adhesive cementation protocol that promotes strong and durable bonding to Y-TZP is essential to ensure high clinical survival rates of zirconia restorations.^39^

Several methods have been developed to strengthen the interface between composite cements and Y-TZP restorations.^66^ Alumina-particle abrasion is a commonly used surface conditioning method to clean and roughen the zirconia surface, modifying its surface energy and wettability.^58^ Currently, the recommended particle abrasion parameters are 50-µm alumina particles and a propulsion pressure of 0.10 to 0.25 .^39^ Previous studies concluded that the bond strength to zirconia was not significantly affected by alumina particle size (small or large grains), despite the different surface roughness values generated.^19,60,62^ Another established method to promote bonding to Y-TZP is tribochemical silica coating followed by silanization. This technique consists of air-abrading the surface with 30- or 110-μm silica-coated alumina particles, aiming to combine micromechanical retention with silica deposition on the zirconia surface, which chemically activates zirconia for a silane-mediated chemical bond.^22,29,58^ However, some laboratory studies have shown that the deposited silica layer is not firmly attached to the hard zirconia surface. As a result, the bond strength to silica-coated zirconia significantly decreases after short artificial aging procedures.^15,25,26,28,42,65^ A previous clinical study also showed that tribochemical silica coating did not promote a stable resin-mediated bond between zirconia inlay-retained fixed dental prostheses and tooth structure.^47^

Different coupling agents have been developed to promote stronger chemical interactions to Y-TZP. These bond-promoting agents contain specific functional monomers which are able to chemically attach to zirconia hydroxyl groups.^37,52,65^ The acidic functional monomer metacryloyloxydecyl dihydrogen phosphate (10-MDP) is the typical phosphate monomer incorporated into the primers. Clinical and in-vitro studies have shown that a durable composite cement bonding to zirconia may be achieved when 10-MDP-containing primers are used after alumina-particle abrasion.^4,30,10,14,50^ A large variety of ceramic primers is available to clinicians, making it difficult to find the most effective product to bond composite cement to zirconia crowns and bridges,^2^ with some clinical studies showing successful outcomes.^24^ Although the use of 10-MDP-containing composite cements to bond alumina-blasted zirconia to tooth structure has shown positive outcomes,^10,23^ the cementation strategy of ceramic restorations generally involves two, three or more steps, making this procedure technique-sensitive and not user-friendly.^1^ In order to overcome these challenges, manufacturers have developed coupling agents with multiple functional components, including 10-MDP and silane, which may be recommended for the cementation of both silicate and high-crystalline-content ceramics. This class of bonding agents, called “universal bonding agents” or “universal adhesives” represents the latest generation of adhesives on the market and combines primer monomers with the components of the adhesive resin. Therefore, although composition varies between different manufacturers, universal bonding agents are considered multicomponent products, which combine phosphate-based monomers, silane, methacrylate monomers and fillers. For the sake of definition in the present study, “universal bonding agents” or “universal adhesives” will be represented by single-bottle, no-mix adhesives used with any bonding strategy and which are recommended for the bonding of ceramic-based and composite-based indirect restorative materials.^1,45,66^ A meta-analysis of in-vitro studies has concluded that universal adhesives may be successful in luting both zirconia and composite-based restorations.^14^ However, it is not clear if the silane molecule in the multicomponent product is capable of chemically bonding to the silica film deposited on the zirconia surface after tribochemical coating.

There are no clinical trials evaluating the effect of universal adhesives on composite-mediated zirconia bonding. The existing literature in the field lacks a compilation of data evaluating the effect of multicomponent systems on composite-mediated bonding to zirconia, so that these materials can be precisely recommended for clinical procedures. Thus, the aim of this study was to perform a systematic review of the literature and analyze bond strength data to evaluate whether universal adhesives are a suitable substitute for phosphate/silane-based primers or phosphate-based primers on composite-mediated bonding to zirconia.

## Material and Methods

This systematic review and meta-analysis was performed in accordance with the PRISMA (Preferred Reporting Items for Systematic Reviews and Meta-Analyses – PRISMA 2020) guidelines.^43^ Additionally, methodological details were registered in Open Science Framework (DOI number 10.17605/OSF.IO/P2GE4). The associated research question set for the development of this study was: are universal adhesives a reliable alternative to phosphate/silane-based primers or phosphate-based primers on composite-mediated bonding to zirconia?

All steps of the systematic review were performed by two trained individuals. For each step, reviewers used 10 peer-reviewed, published studies to perform evaluator calibration. Any inter-examiner disagreement was resolved by other authors.

### Eligibility Criteria

#### Inclusion criteria

- The studies evaluated composite-mediated bonding to zirconia using universal adhesives and phosphate/silane based-primer or phosphate-based primer were included.
- Zirconia surfaces blasted with 30-µm to 150-µm alumina or silica-coated alumina particles.
- The studies analyzed mean and standard deviation of bond strength data in MPa using shear, microshear, tensile, or microtensile tests.

#### Exclusion criteria

- The studies used exclusively self-adhesive composite cements to bond to zirconia.
- Other types of surface treatment such as plasma, laser, or glass infiltration were used.
- Exclusively experimental materials were used.
- Studies not published in English.
- Letters to the editor, comprehensive reviews, and conference abstracts.

### Information Sources and Search Strategy

Three distinct electronic databases (PubMed/Medline, Scopus and ISI Web of Science) were accessed to conduct electronic and systematic searches. These systematic searches were performed by two of the authors who were previously calibrated for database searching. The last search in the databases was conducted in August 2021. The following MeSH (Medical Subject Headings), “text words” and their combinations were used: ‘zirconia’ OR ‘zirconia ceramic’ OR ‘yttria stabilized tetragonal zirconia’ OR ‘Y-TZP’ OR ‘polycrystalline’ AND ‘universal adhesive’ OR ‘universal adhesives’ OR ‘multi-mode bonding agent’ OR ‘multimode bonding agent’ using advanced option. In addition, searches were conducted by reading the reference lists from all selected studies to identify other possible manuscripts.

### Selection Process

Titles and abstracts were included in the Mendeley reference manager to remove duplicates. Afterwards, titles and abstracts were screened, read, and categorized in accordance with the defined selection criteria. Two authors independently conducted a categorization process and later compared their findings. Possible disagreements in this process were discussed with other authors. Potentially eligible manuscripts were downloaded from databases in full-text version for further analysis, data extraction, and risk of bias assessment.

### Data Collection

To extract the most relevant methodological data from selected studies, two authors used a standardized collection form. The extracted data included were author name, publication year, commercial reference of monolithic zirconia, presence or absence of abrasion treatment (air abrasion with alumina and/or silica-coated alumina particles), type of primer (phosphate and/or phosphate-silane primer) and/or universal adhesive (commercial reference), method for analysis of bond strength, aging process, mean and standard deviation of the bond strength results. Some manuscripts presented data in graphic format and, in those cases, the corresponding authors were contacted via e-mail to provide numeric values.

### Study Risk of Bias Assessment

The risk of bias was evaluated by two authors based on previous systematic reviews of in-vitro studies related to dental materials^30,48,55^ and the Cochrane Collaboration’s tool.^20^ Aspects such as randomization, sample size calculation, comparability among groups, detailed information regarding measurements, proper statistical analysis, adherence to manufacturer’s instructions, and single and/or blinded operator among included studies, were analyzed. The risk of bias was categorized as high, low, or unclear, based on how and whether each of the mentioned items were reported.

### Data Analysis

The meta-analysis of the studies that met the inclusion criteria was conducted using special software (Review Manager, version 5.4, Cochrane Collaboration). A random-effects model was used when high heterogeneity (I^2^ > 50%) was detected. A fixed-effects model was also used as needed. A pooled-effect estimate was obtained by comparing the standardized mean difference between bond strengths obtained using the phosphate or phosphate/silane-based primer or universal adhesive. For all analyses, a p-value = 0.05 was considered statistically significant. Subgroup analyses were also performed to assess different types of air-abrasion treatment (no abrasion, air abrasion with 45-µm alumina or with 30-µm silica-coated alumina particles) and aging conditions (short-term/no aging, or long-term/static or dynamic aging). Static aging consisted of water storage and dynamic aging comprised thermocycling with or without additional water storage.

## Results

### Study Selection

**Fig 1:**
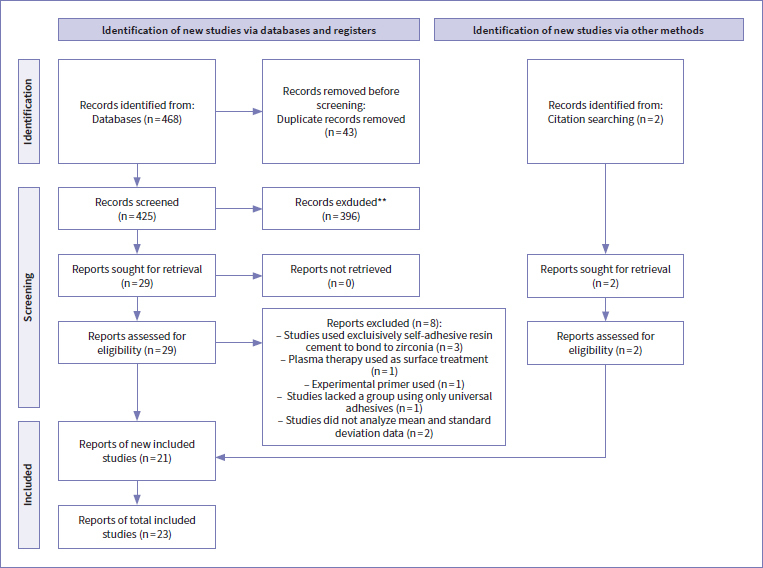
PRISMA 2020 flow diagram summarizing identification and selection process.

The PRISMA flowchart that summarizes the search strategy is shown in Fig 1. A total of 468 publications were selected from all databases, of which 43 were eliminated due to duplication. After analysis of title and abstract, 396 reports were excluded. To screen other potentially eligible studies, complementary database searches filtered by key author/co-author name and reference lists of the included studies were performed, and 2 additional manuscripts were found. Thus, 31 potentially eligible studies were assessed by full-text reading. According to inclusion and exclusion criteria, 8 studies were excluded at this stage (Fig 1). In total, 23 studies were included in the qualitative and quantitative analysis.

### Study Characteristics

Tables 1 shows the main methodological aspects of the studies included in this systematic review. All manuscripts were published between 2014 and 2021. The most frequently tested zirconia reported was IPS e.max ZirCAD (Ivoclar Vivadent; Schaan, Liechtenstein) (n = 4, 18.2%), followed by Lava Zirconia (3M Oral Care; St Paul, MN, USA) (n = 3, 13.6%) and Vita In-Ceram YZ (Vita Zahnfabrik; Bad Säckingen, Germany) (n = 3, 13.6%). Nineteen studies evaluated airborne alumina-abraded zirconia surfaces (81.8% of total) and six studies reported the use of 30-µm silica-embedded alumina particles. For both particles (alumina and silica-embedded alumina), the most frequently reported airborne-particle abrasion conditions were: 0.25 MPa for 15 s at a distance of 10 mm. In terms of phosphate-containing primers, the 10-MDP-containing Z-Prime Plus (Bisco; Schaumburg, IL, USA) was the most frequently used (n = 14, 60.8%), and for phosphate/silane-containing primers, Monobond Plus (Ivoclar Vivadent; Schaan, Liechtenstein) was the most frequently reported (n = 5, 21.7%). MZ primer (Angelus; Londrina, Paraná, Brazil) was excluded from the meta-analysis because it was not possible to find any information confirming the existence of phosphate-based monomers. Four universal adhesives were reported in the studies included, as follows: Scotchbond Universal (3M Oral Care) (n = 20, 86.9%), All Bond Universal (Bisco) (n = 6, 26.1%), Clearfil Universal Bond (Kuraray Noritake; Okayama, Japan) (n = 5, 21.7%), Futura Bond U (Voco; Cuxhaven, Germany) (n = 1, 4.5%). The composition and manufacturers of all primers and adhesives included in this study are shown in Table 2. The composite-mediated zirconia bond strength tests reported were shear (n = 14, 60.8%), microshear (n = 4, 17.4%), tensile (n = 4, 17.4%), and microtensile (n = 1, 4.3%).

**Table 1:** Main methodological data from included studies

Authors, year	Sample size (n)	Zirconia	Alumina-particle abrasion	Silica coating	Primer (phosphate- and/or silane-containing)	Universal adhesive	Resin cement/Resin composite	Bond strength test	Aging
Amaral et al, 2014^2^	15	Vita In-Ceram YZ (Vita Zahnfabrik)	*Yes/No	Yes	Z-Prime Plus, AZ Primer, Monobond Plus	Scotchbond Universalø	Variolink II	μTBS**	Thermocycling
Seabra et al, 2014^51^	10	Lava Frame Zirconia (3M Oral Care)	Yes	No	Z-Prime Plus	Scotchbond Universal, All-Bond Universal	Filtek Z250	SBS***	No
Kim et al, 2015^26^	10	Zirconia Cercon base (DeguDent)	No	No	Alloy Primer	Scotchbond Universal	RelyX ARC	μSBS****	Thermocycling
Pereira et al, 2015^45^	10	Lava Zirconia (3M Oral Care)	Yes/No	No	Alloy Primer, Metal Zirconia Prime, Monobond Plus, Signum Zirconia Bond, Z Prime Plus	Scotchbond Universal	RelyX ARC	SBS	Water storage
Bomicke et al, 2016^6^	10	IPS e.max ZirCAD (Ivoclar Vivadent)	Yes	Yes	Clearfil Ceramic Primer	Scotchbond Universal	RelyX Ultimate	TBS*****	Water storage and/or thermocycling
Lopes et al, 2016^32^	24	Prettau Zirconia (Zirkonzahn)	Yes	No	Signum Zirconia Bond I + II, Z-Prime Plus	Scotchbond Universal	Duo-Link Dual	μSBS	Water storage
Passia et al, 2016^44^	16	IPS e.max ZirCAD (Ivoclar Vivadent)	Yes	No	Monobond Plus	Scotchbond Universal	Multilink Automix, RelyX Ultimate	TBS	Water storage and thermocycling
Xie et al, 2016^65^	10	YTZP/Everest ZS-Ronde (KAVO)	Yes	Yes	Z-Prime Plus	Scotchbond Universal, Clearfil Universal Bond, All-Bond Universal	Variolink N	SBS	Thermocycling
Zhao et al, 2016^73^	8	Lava Zirconia (3M Oral Care)	Yes	No	Z-Prime Plus	Scotchbond Universal	RelyX Ultimate	SBS	Water storage and thermocycling
Elsayed et al, 2017^16^	8	Zirconia Zenostar T (Wieland)	Yes	No	Monobond Plus	Scotchbond Universal, All Bond Universal	RelyX Ultimate, Duo Link Universal	TBS	Water storage and /or thermocycling
Llerena-Icochea et al, 201731	4	IPS e.max ZirCAD (Ivoclar Vivadent)	No	No	Signum Zirconia Bond	Scotchbond Universal	RelyX Ultimate	SBS	No
Araújo et al, 2018^3^	30	Lava Zirconia (3M Oral Care)	No	Yes	EspeSil	Scotchbond Universal	RelyX Ultimate	SBS	Water storage
Butler et al, 2018^9^	10	NeZr (Sagemax Bioceramic)	No	Yes	Z-Prime Plus	All-Bond Universal, ScotchBondUniversal	Duo Link cement	SBS	No
Moura et al, 2018^35^	10	Vita In-Ceram- YZ2000 (Vita Zahnfabrik )	Yes	No	Monobond Plus	Scotchbond Universal	RelyX Ultimate, Multilink N	SBS	Water storage and/or thermocycling
Sharafeddin et al, 2018^52^	10	Zirconia ceramic (DDcube X2 Dental Direct Materials)	Yes	No	Z-Prime Plus	All- Bond Universal	Variolink N	SBS	No
Silva et al, 2018^53^	15	Zirconia Fit Plus (Talmax)	Yes	No	Z Prime Plus, Signum Zirconia Bond	Scotchbond Universal	Empress Direct	TBS	No
Yang et al, 2018a^67^	10	Zirconia Y-TZP (Shenzhen Santo)	Yes	No	Z-Prime Plus, Clearfil Ceramic Primer	Scotchbond Universal, Clearfil Universal Bond	RelyX Veneer	SBS	Thermocycling
Yang et al, 2018b^68^	11	Lava Zirconia Plus (3M Oral Care)	Yes	No	Z-Prime Plus, Clearfil Ceramic Primer	Scotchbond Universal, Clearfil Universal Bond	RelyX Veneer	SBS	Water storage and thermocycling
Dos Santos et al, 2019^15^	10	IPS e.max ZirCAD (Ivoclar Vivadent)	Yes/No	No	Z-Prime Plus	All Bond Universal, Scotchbond Universal	Z350 XT	μSBS	No
Lima et al, 2019^28^	10	Lava Zirconia Plus (3M Oral Care)	Yes	Yes	RelyX Ceramic Prime, Clearfil Ceramic Primer, Alloy Primer	Clearfil Universal Bond	RelyX Ultimate	μSBS	Water storage
Salem et al, 2019^48^	8	Starceram Z (H.C. Starck)	Yes	No	Z-PRIME Plus, Clearfil Ceramic Primer Plus	Scotchbond Universal	RelyX Ultimate, Duo-Link	SBS	Thermocycling
Zakavi et al, 2019^72^	10	Vita In-Ceram YZ (Vita Zahnfabrik)	Yes	No	Z-Prime Plus primer	Futurabond U adhesive, Clearfil universal bond adhesive	Valux Plus	SBS	Thermocycling
Moradi et al, 2021^34^	10	Incoris Sirona (Dentsply)	Yes/No	Yes	Bis-Silane, Z-Prime Plus	Scotchbond Universal	Duo-link	SBS	Thermocycling

*Both conditions, with and without alumina abrasion, were analyzed; ** μTBS: microtensile bond strength; ***TBS: tensile bond strength; ****μSBS: microshear bond strength; *****SBS: shear bond strength. ø This universal adhesive is also marketed as Single Bond Universal.

**Table 2:** Composition and manufacturers of primers and universal adhesives reported in the included studies

Material	Composition	Manufacturer	Concentration of phosphate monomer % by weight
All-Bond Universal	10-MDP, bis-GMA, HEMA, ethanol	Bisco; Schaumburg, IL, USA	5–25
Alloy Primer	10-MDP, VBATDT, acetone	Kuraray Noritake; Tokyo, Japan	<5
AZ Primer	6-MHPA5, acetone	Shofu; Kyoto, Japan	<5
Clearfil Ceramic Primer	10-MDP, 3-trimethoxysilylpropyl methacrylate, ethanol	Kuraray Noritake	<5
Clearfil Ceramic Primer Plus	10-MDP, 3-trimethoxysilylpropyl methacrylate, ethanol	Kuraray Noritake	<5
Clearfil Universal Bond	10-MDP, bis-GMA, HEMA, hydrophilic aliphatic dimethacrylate, colloidal sílica, dl-camphorquinone, silane coupling agent, accelerators, initiators, water, ethanol	Kuraray Noritake	–
EspeSil	Ethyl alcohol, 3-methacryloxypropyl trimethoxysilane, methyl ethyl ketone	3M Oral Care; St Paul, MN, USA	
Futurabond U	Bis-GMA, HEDMA, HEMA, UDMA, acidic adhesive monomer, catalyst	Voco; Cuxhaven, Germany	5–10
Metal Zirconia Primer	Phosphonic acid acrylate, dibenzoyl peroxide, methylisobutylketone, tert-butyl alcohol	Ivoclar Vivadent; Schaan, Liechtenstein	2.5–10
Monobond Plus	10-MDP, ethanol, 3-trimethoxysilylpropyl methacrylate, sulphide methacrylate	Ivoclar Vivadent	<2.5
RelyX Ceramic Primer	Ethyl alcohol, water, Methacryloxypropyltrimethoxysilane	3M Oral Care	
Scotchbond Universal	10-MDP, HEMA, Bis-GMA, Vitrebond copolymer, silane, dimethacrylate resins, ﬁllers, initiators, ethanol	3M Oral Care	10–20
Signum Zirconia Bond I	10-MDP, acetone, acetic acid	Heraeus Kulzer; Hanau, Germany	0–5
Signum Zirconia Bond II	Methyl methacrylate, diphenyl(2,4,6- trimethylbenzoyl) phosphine oxide	Heraeus Kulzer	
Z-Prime Plus	Bis-GMA, HEMA, 10-MDP, ethanol	Bisco; Schaumburg, IL, USA	1–5

10-MDP: 10-methacryloyloxydecyl dihydrogen phosphate; bis-GMA: bisphenol A diglycidylmethacrylate; HEMA: 2-hydroxyethyl methacrylate; VBATDT: 6-(4-vinylbenzyl-N-propyl)amino-1,3,5-triazine-2,4-dithione; 6-MHPA: 6-methacryloxyhexyl phosphonoacetate; HEDMA: 1, 6-hexanediyl bismethacrylate; UDMA: urethane dimethacrylate.

### Risk of Bias

**Fig 2:**
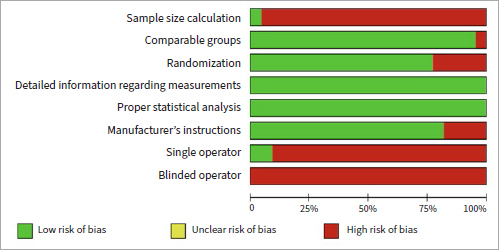
Distribution of risk of bias among the studies included according to pre-established criteria.

The majority of the included reports presented a high risk of bias only in terms of sample size calculation, single and blinded operator. A low risk of bias was observed regarding comparable groups, randomization, detailed information regarding measurements, proper statistical analysis, and manufacturers’ instructions (Fig 2).

### Results of Syntheses

**Fig 3:**
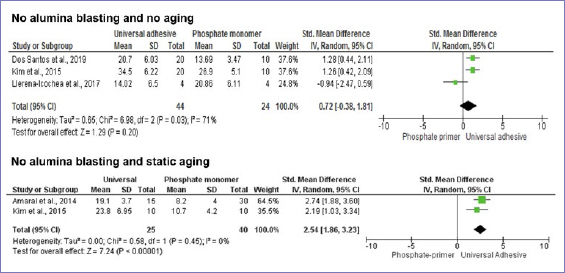
Forest plots summarizing bond strengths to zirconia when phosphate-based primers and universal adhesives were applied: without alumina-particle abrasion, tested without aging; without alumina-particle abrasion, tested after static aging.

**Fig 4:**
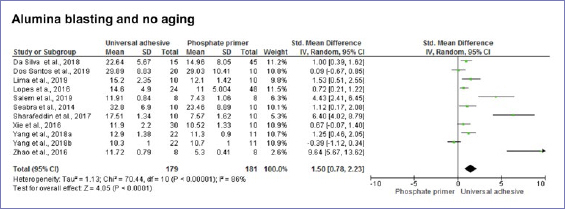
Forest plots summarizing bond strengths to zirconia when phosphate-based primers and universal adhesives were applied: after alumina-particle abrasion, tested without aging.

**Fig 5:**
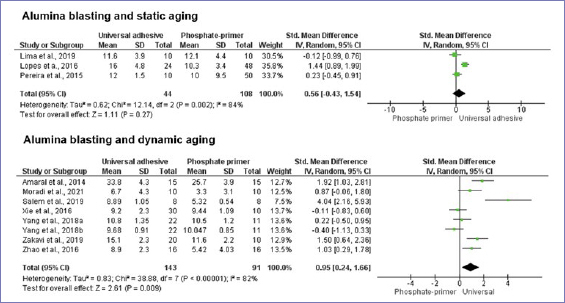
Forest plots summarizing bond strengths to zirconia when phosphate-based primers and universal adhesives were applied: after alumina-particle abrasion, tested after static and dynamic aging.

**Fig 6:**
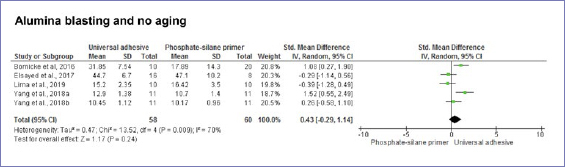
Forest plots summarizing bond strengths to zirconia when phosphate/silane-based primers and universal adhesives were applied: after alumina-particle abrasion, tested without aging.

**Fig 7:**
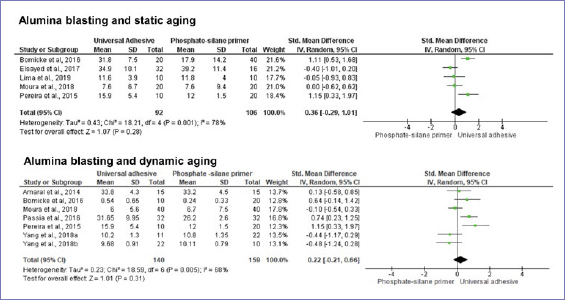
Forest plots summarizing bond strengths to zirconia when phosphate/silane-based primers and universal adhesives were applied: after alumina-particle abrasion, tested after static and dynamic aging.

According to the random-effect model, bond strength results between zirconia and resin-based cement were similar when universal adhesives or phosphate-based primers were compared, considering no alumina-particle abrasion and no aging (p = 0.20) (Fig 3). Nonetheless, after dynamic aging, the use of universal adhesives significantly increased the composite-mediated bond strength to zirconia (Fig 3). When the zirconia surface was blasted with alumina, the application of universal adhesives improved bond strength at both timepoints: initially and after dynamic aging (Figs 4 and 5). Similar bond strengths (p = 0.2) were observed when universal adhesives or phosphate-based primers were used to bond to zirconia and static aging was considered (Fig 5). In addition, there was no difference in bond strength between phosphate/silane-containing primers and universal adhesives after alumina-particle abrasion, regardless of storage condition (Figs 6 and 7).

**Fig 8:**
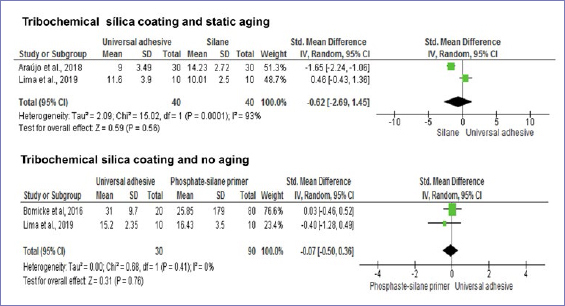
Forest plots summarizing bond strengths to zirconia when: silane-based primer and universal adhesives were applied after tribochemical silica coating and tested after static aging, and phosphate/silane-based primer and universal adhesives were applied after tribochemical silica coating and tested without aging.

**Fig 9:**
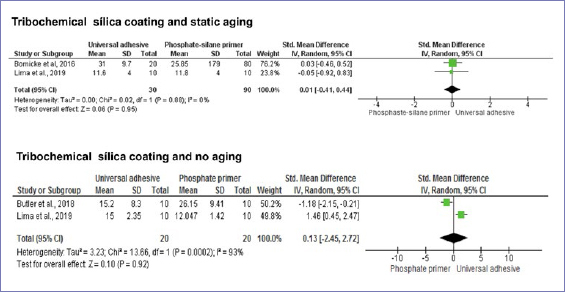
Forest plots summarizing bond strengths to zirconia when: phosphate/silane-based primer and universal adhesives were applied after tribochemical silica coating and tested after aging, and phosphate-based primer and universal adhesives were applied after tribochemical silica coating and tested after static aging.

Figures 8 and 9 show the bond strengths when zirconia was blasted with silica-coated alumina particles. The bond strengths to zirconia were similar when universal adhesives or conventional zirconia-specific primers were compared, regardless of aging conditions.

## Discussion

The clinical survival of zirconia restorations is largely affected by the cementation protocol, operator skills, patient compliance, and quality of the manufacturing process. This systematic review and meta-analysis of in-vitro studies was carried out to identify the effect of the chemical treatment of zirconia on its bond strength. The effect of different factors such as type of bond strength test and artificial aging (static or dynamic) on the composite-mediated zirconia bond was also evaluated.

Considering bond strength tests, the shear bond strength test was the most frequently reported (n = 13, 54.2%). Other systematic reviews reported similar results, confirming that shear tests have been widely used to assess the bond strength of composite cements to ceramics.^21,41,57^ A previous study reported that shear bond strength tests may be less sensitive in indicating bond strength differences to zirconia in comparison to microshear tests and tensile or microtensile tests, because the shear bond strength methods may not directly test the bonded interface.^28^ The heterogeneity of the stresses applied to the bonded interface and also the occurrence of cohesive failures in both substrates evaluated are some of the reasons given by researchers.^29,54,69^ Currently, consensus is lacking about which test method is the most appropriate to evaluate bond strength to zirconia,^28^ as reflected by the variety of studies reporting the use of different bond strength tests in the literature.^14,40^ As such, the bond strength data reported in the studies included in this review was highly heterogeneous (Figs 3 to 9). Another factor analyzed in this systematic review was the aging method: static or dynamic. The most frequent aging methods reported in the studies included were water storage (static aging),^3,7,17, 28,34,37,47^ and thermocycling (dynamic aging) with or without water storage.^2,44,26,34,48,67,68,72,73^ Six studies did not use any aging method.^9,15,31,51-53^ The interface between the coupling agent and zirconia may undergo hydrolytic degradation of the interfacial components, compromising bond strength in the long-term. In addition, temperature changes during thermocycling may expedite fatigue at the bonded interface, resulting in further degradation.^58^ Using universal adhesives for bonding to zirconia seems to induce a higher rate of degradation at the interface after aging.^17,28^ In contrast, many studies showed that using phosphate-based primers results in bond strengths that do not decrease after aging.^20,28,40,58^

Some studies have indicated that the combination of chemical and mechanical pre-treatments is fundamental to achieve a durable composite/zirconia interface.^5,6,28,66^ Surface abrasion (mechanical treatment with alumina-particle abrasion) has been applied to the zirconia surface to increase surface roughness, allowing micromechanical interlocking of the cement into the surface irregularities.^60^ Most of the included in-vitro studies analyzed airborne alumina-particle-abraded zirconia surfaces (n = 19, 79.2%), using 50-µm alumina particles at 0.25 MPa for 15 s at a distance of 10 mm. Previous studies have described a complex surface topography associated with high bond strengths when zirconia is abraded with 50-µm alumina particles.^5,6,66^ As previously mentioned, alumina-particle abrasion not only promotes micromechanical interlocking but also increases the surface area for bonding.

The studies included in this review reported either no mechanical treatment or one of two types of surface abrasion to zirconia: particle abrasion with alumina or with silica-coated alumina particles. When no mechanical treatment was reported, universal adhesives showed similar or better bond strength to zirconia than did phosphate-based primers, both initially and after artificial aging (Fig 3). Similar results were observed when zirconia was abraded with alumina particles (Figs 4 and 5). The effectiveness of universal adhesives to bond to zirconia when compared to phosphate-based primers can be explained by the chemical composition of the universal adhesives. All universal adhesives evaluated in this metanalysis contained 10-MDP as the main functional component, except for Futurabond U (Voco; Cuxhaven, Germany), which has a specific acidic monomer (Table 2). The 10-MDP molecule reacts chemically with the zirconium oxide surface via hydrogen bonds, or via the ionic interaction between the P-OH and Zr-OH groups, or between P-O− and partially positive Zr.^37^ Other components present in the chemistry of the universal adhesives are methacrylate and dimethacrylate monomers (HEMA, bis-GMA, and UDMA), Vitrebond copolymer, silane, and fillers. It is possible that these chemical components improve the bond strength to zirconia by increasing zirconia surface wettability and providing a moisture-stabilizing effect.^12,26,28^ Consequently, the interface formed between 10-MDP and zirconia may become more hydrophobic and stable over time.^13^

Several studies showed that when 10-MDP-based primers are associated with alumina-particle abrasion to mediate bonding to zirconia, high and stable values are observed.^22,24,27,28,49,50^ Most of phosphate-based primers included in this meta-analysis contained 10-MDP as the main functional component. The meta-analysis results showed that universal adhesives provided better bond strength to zirconia than did phosphate-based primers under similar experimental conditions. A stronger bond to zirconia can be achieved when the commercial dental primer or adhesive has a high purity grade and high concentration of 10-MDP monomer.^40^ Overall, universal adhesives have higher wt% of 10-MDP than do phosphate-based primers. Some studies have shown that the chemical affinity of 10-MDP to zirconia is optimal only when using a solution of 10 wt% 10-MDP.^11,63^ Table 2 shows the chemical components and wt% of phosphate monomers of all bonding agents reported in the studies included in this meta-analysis, according to manufacturers’ information. Most of the studies evaluated the performance of Scotchbond Universal (3M Oral Care), which has 10-20 wt% 10-MDP (Table 2). Only one universal adhesive (Clearfil Universal Bond, Kuraray Noritake) does not disclose specific information about its 10-MDP concentration. Studies revealed that this specific system had a low percentage of P-O-Zr bonds and low bond strengths in comparison to 10-MDP-based primers.^28,33^ Thus, the extension of findings of this systematic review and meta-analysis to all universal adhesives should be made with caution, due to the chemical variability reported amongst different commercial products. This indicates that the performance of primers and adhesives on bonding to zirconia may be anticipated by their chemical composition, so that decision-making for clinicians is safer and simpler.

Phosphate/silane-containing primers are available on the market for the cementation of zirconia and glass-ceramic restorations. According to the results of this systematic review and meta-analysis, those primers showed similar bond strengths to zirconia when compared to universal adhesives for all study conditions considered here (alumina-particle abrasion and aging) (Figs 6 and 7). All phosphate/silane-containing primers reported in the included studies contain silane and 10-MDP. This silane/10-MDP combination may have improved resin-zirconia bonding by improving surface wettability, which allowed a better flow of the composite cement over the zirconia surface.^57,70^ Furthermore, the methacrylate groups of silane and 10-MDP molecules may form crosslinks between them as well as siloxane bonds with the OH groups of the zirconia surface.^14,21^ Therefore, it seems that the combination of 10-MDP and silane promote a synergistic effect on bonding to zirconia.

All primers studied (phosphate, phosphate-silane, and silane-based primers) showed similar bond strengths when compared to universal adhesives after tribochemical coating (Figs 8 and 9). This finding was certainly surprising, since the covalent bond between silane and the silica film deposited on the zirconia surface is expected to be stronger, promoting higher bond strength for silane-based primers than phosphate, phosphate/silane-based primers, and universal adhesives, as previously reported in another systematic review.^20^ It has been demonstrated that the silane-silica interaction is more thermodynamically stable under hydrolytic conditions than is the phosphate-zirconia interaction.^66^ One fact that may explain the similar values between different materials is that the silica particles are only weakly attached to the zirconia surface and may detach easily.^24,28,69^ Therefore, even if a strong chemical interaction occurred between silane and silica, the silica was still poorly attached to the surface of zirconia, being the weak link that compromised bond strength. In addition, some studies have shown that the silane contained in both phosphate/silane-based primers and universal adhesives may not be stable and, consequently, is inactive after a short time, jeopardizing the formation of silane-silica chemical bonds.^37,72^ Thus, it is possible that the bond strength between phosphate/silane based-primers or universal adhesives and silica-blasted zirconia is provided mainly by the phosphate-zirconia chemical interaction.

The studies included in this systematic review and meta-analysis evaluated resin-mediated bonding to zirconia with (n = 17 studies) or without (n = 6 studies) artificial aging. Seven studies used thermocycling alone (from 2500 to 37,500 thermocycles), 7 studies used water storage (30 days to 6 months) and 6 studies combined thermocycling and water storage (5000 to 37,500 thermocycles and 30 days to 6 months). Bond strengths were significantly affected by aging in 12 studies. A previous study analyzing the effect of chemical and mechanical treatments on bond strength to zirconia showed that surface treatment with 10-MDP-containing materials promoted the highest bond strengths, regardless of the aging conditions.^57^ Although in-vitro aging cannot accurately reproduce the challenges that dental restorations are exposed to in the oral environment, they certainly allow for an estimation of the hydrolytic stability of the chemical interactions. Immediate bond strengths, as reported by 8 of the studies included in this review, should be viewed with caution, but they provide an indication of the interactions between adherent and adhesive. Therefore, promising in-vitro results should be further investigated by prospective, retrospective, or randomized clinical trials with a follow-up of at least 5 years and different prosthetic designs.^4^

Based on the results of this systematic review, the bonding performance of universal adhesives was similar to or better than when compared to the phosphate-based primers and/or silane-based primers analyzed, regardless of surface abrasion procedures and simulated aging parameters. The risk of bias analysis showed that most of the in-vitro studies included did not report sample size calculation or proper information about the operator. Additionally, a high heterogeneity in the meta-analysis was observed, probably because of the methodological differences between the studies. Thus, these results should also be interpreted with caution. As a future step, the findings of the present study might be used to design clinical trials that would validate the data obtained in this systematic review.

## Conclusion

In spite of the heterogeneous bond strength data and the various bond strength tests reported, the meta-analysis of the collected data revealed that universal adhesives may be effective materials to replace phosphate-based primers and/or silane-based primers in bonding to zirconia restorations at both simulated aging times, short- or long-term. However, these results should be carefully considered for the different clinical applications due to the chemical variability observed between different systems. The observations of the present study also indicate that clinical trials would add important information to the body of evidence, due to the heterogeneity of the studies included and reviewed in this meta-analysis.

## Clinical relevance

Universal adhesives may be a reliable alternative to simplify the clinical luting of zirconia-based restorations without compromising bond strength.
